# Expression Analysis of microRNAs in Peripheral Blood Mononuclear Cells in Type 2 Diabetes

**DOI:** 10.3390/biom16101503

**Published:** 2026-10-15

**Authors:** Yanina Timasheva, Olga Kochetova, Vitaly A. Markelov, Ksenia V. Danilko, Tatyana M. Kochetova, Diana Avzaletdinova, Gulnaz F. Korytina, Zhanna Balkhiyarova, Arie Nouwen

**Affiliations:** 1 Institute of Biochemistry and Genetics, Ufa Federal Research Centre, Russian Academy of Sciences, 450054 Ufa, Russia; ovkochetova@bashgmu.ru (O.K.); marckelow.vitalick2017@yandex.ru (V.A.M.); gfkorytina@bashgmu.ru (G.F.K.); 2 Department of Medical Genetics and Fundamental Medicine, Faculty of General Medicine, Bashkir State Medical University, 450008 Ufa, Russia; 3 Department of Biology, Faculty of Stomatology, Bashkir State Medical University, 450008 Ufa, Russia; 027620666357@bashgmu.ru; 4 Laboratory of Microfluidic Technology, Institute of Fundamental Medicine, Bashkir State Medical University, 450008 Ufa, Russia; 5 Laboratory of Cell Cultures, Institute of Fundamental Medicine, Bashkir State Medical University, 450008 Ufa, Russia; kvdanilko@bashgmu.ru; 6 Department of Endocrinology, Faculty of General Medicine, Bashkir State Medical University, 450008 Ufa, Russia; dshavzaletdinova@bashgmu.ru (D.A.); z.balkhiiarova@surrey.ac.uk (Z.B.); 7 Section of Statistical Multi-Omics, Department of Clinical & Experimental Medicine, School of Biosciences & Medicine, University of Surrey, Guildford GU2 7XH, UK; 8 Department of Psychology, Middlesex University, London NW4 4BT, UK; a.nouwen@mdx.ac.uk

**Keywords:** type 2 diabetes, obesity, microRNAs, microRNA expression

## Abstract

MicroRNAs (miRNAs) are involved in the regulation of glucose metabolism and inflammatory processes associated with type 2 diabetes (T2D) and have potential as biomarkers and therapeutic targets. We analysed the expression of miR-155-5p, miR-141-3p, miR-15b-5p, miR-18a-5p, and miR-379-5p in peripheral blood mononuclear cells (PBMCs) from 79 patients with T2D and 88 non-diabetic controls. Differential expression analysis revealed significant downregulation of miR-15b-5p, miR-155-5p, and miR-18a-5p, and significant upregulation of miR-379-5p in T2D patients. Among the individual miRNAs, miR-15b-5p showed the highest ability to distinguish between T2D and controls (AUC = 0.82, 95% CI 0.76–0.89, *p* = 0.001). A model combining the four differentially expressed miRNAs (miR-155-5p, miR-15b-5p, miR-18a-5p, and miR-379-5p) with age, sex, and BMI had an AUC of 0.98, compared with 0.93 for age, sex, and BMI alone (*p* = 0.004, paired DeLong test); the optimism-corrected AUC was 0.97, although the bootstrap procedure did not account for optimism arising from miRNA selection. Functional enrichment analysis identified MAPK, Hippo, FoxO, insulin, AMPK and TGF-β signalling as consistently enriched across analyses based on experimentally supported and predicted miRNA targets.

## 1. Introduction

Diabetes is among the most prevalent chronic diseases worldwide, with incidence rising alongside lifestyle-driven increases in obesity, the principal driver of type 2 diabetes (T2D) [1]. T2D accounts for approximately 90% of all diabetes cases [1]. Although considerable progress has been made in understanding its molecular basis, reliable molecular biomarkers that provide information beyond established clinical measures remain limited. MicroRNAs (miRNAs) have attracted considerable interest because of their regulatory functions and stability in biological samples. However, no reproducible miRNA expression signature has yet been established for T2D, and the biological significance of many reported associations remains unclear.

MicroRNAs are non-coding RNAs of approximately 19–22 nucleotides that regulate gene expression primarily at the post-transcriptional level. They are involved in numerous processes relevant to T2D, including insulin signalling, glucose metabolism, inflammation, and pancreatic β-cell function [2]. Their stability in tissues and biological fluids has also made them attractive biomarker candidates [3]. However, because miRNA expression is highly cell- and tissue-specific, the biological source analysed is likely to influence the observed expression profile and its biological interpretation. Combining miRNA expression data with bioinformatic analyses of target genes and signalling pathways may therefore provide a more informative view of the molecular processes involved in T2D [4].

Most studies have examined circulating miRNAs in serum, plasma or whole blood, and numerous candidate biomarkers have been proposed [3,5,6,7]. However, reported findings remain inconsistent, probably owing to differences in study populations, analytical methods and the biological material analysed [8,9]. In contrast, relatively few studies have investigated miRNA expression in peripheral blood mononuclear cells (PBMCs). PBMCs are of particular interest because chronic low-grade inflammation is a key feature of T2D, and immune cells contribute to insulin resistance through altered inflammatory signalling. Characterising miRNA expression in PBMCs may therefore provide insight into regulatory mechanisms operating within these cells while also identifying candidate molecular markers associated with T2D.

To select candidate miRNAs, we combined a comprehensive literature review with analysis of publicly available NCBI Gene Expression Omnibus (GEO) datasets. This approach identified five miRNAs–miR-155-5p, miR-141-3p, miR-15b-5p, miR-18a-5p, and miR-379-5p–that have been repeatedly implicated in T2D or its complications (Table S1). We then investigated their expression in PBMCs and integrated the experimental findings with target identification and pathway enrichment analyses to assess their potential biological significance.

The aim of this study was to characterise the expression of selected miRNAs in PBMCs from patients with T2D and non-diabetic controls and to assess their associations with T2D status, clinical characteristics and potential biological functions, as well as their incremental discriminatory value beyond major demographic and anthropometric covariates.

## 2. Materials and Methods

### 2.1. Study Group

The study protocol was approved by the Institutional Ethics Committee (Protocol No. 17, 7 December 2012), and written informed consent was obtained from all participants. This study included 79 individuals with T2D and 88 non-diabetic controls. Patients with T2D were recruited at the primary healthcare level. Eligibility was established based on a comprehensive clinical evaluation, including medical history, prior diabetes diagnosis, comorbid conditions, and medication use. T2D was diagnosed according to the World Health Organization (WHO) criteria (2013). Arterial hypertension was defined as a blood pressure ≥140/90 mmHg and/or the use of antihypertensive medication. Diabetic nephropathy was diagnosed in patients with diabetes who exhibited a persistent estimated glomerular filtration rate (eGFR) <60 mL/min/1.73 m^2^ and/or albuminuria, defined as an albumin-to-creatinine ratio (ACR) ≥30 mg/g, persisting for at least 3 months. Diabetic neuropathy was diagnosed based on clinical and neurological examination, including assessment of vibration perception at the great toe, temperature sensation, 10-g monofilament testing, and/or nerve conduction studies. Diabetic retinopathy was identified from ophthalmologic evaluations documented in medical records, with fundoscopic findings graded according to the Early Treatment Diabetic Retinopathy Study (ETDRS) classification. Exclusion criteria included other types of diabetes, pregnancy, lactation, and close familial relatedness among participants. The control group comprised 88 individuals without diabetes or prediabetes, defined by fasting plasma glucose < 6.1 mmol/L [10] and HbA1c < 5.7% [11].

### 2.2. Anthropometric Measurements and Biochemical Assays

All participants underwent anthropometric assessment, including measurement of height and body weight. Body mass index (BMI) was calculated as weight (kg) divided by height squared (m^2^). Blood samples were collected after a 12 h overnight fast and 2 h after a meal for assessment of postprandial glucose. HbA1c was measured by high-performance liquid chromatography using an ADAMS A1c HA-8182 analyser (Arkray Inc., Kyoto, Japan). Plasma glucose was measured using the glucose oxidase method (Cobas Integra, Roche Diagnostics GmbH, Mannheim, Germany). Total cholesterol, triglycerides, high-density lipoprotein (HDL) cholesterol, and low-density lipoprotein (LDL) cholesterol were measured by photometric methods (Olympus, Hamburg, Germany).

### 2.3. Selection of Candidate miRNAs

We searched the NCBI Gene Expression Omnibus (GEO) database for human miRNA expression studies involving PBMCs or comparisons between patients with T2D and non-diabetic controls. Two publicly available datasets were examined: GSE75026, to assess miRNA expression in PBMCs, and GSE21321, to assess differential miRNA expression in T2D. We examined the deposited expression data and published results and searched the literature for additional evidence associating individual miRNAs with T2D or its complications. Candidate miRNAs were selected based on evidence of their expression in PBMCs and published associations with T2D or its complications.

### 2.4. PBMC Isolation and Preparation

For miRNA analysis, 4 mL of peripheral venous blood was collected into EDTA-containing tubes and processed within 1 h of collection. PBMCs were isolated by Ficoll-Paque density-gradient centrifugation. Briefly, blood was diluted 1:1 with phosphate-buffered saline (PBS), layered over 3 mL of Ficoll-Paque solution (density, 1.077 g/mL; Cytiva, Marlborough, MA, USA), and centrifuged at 420× *g* for 30 min at room temperature. The PBMC layer was collected, washed twice with PBS, and homogenised in 1 mL of TRIzol reagent (Thermo Fisher Scientific, Waltham, MA, USA) for subsequent RNA extraction.

### 2.5. RNA Extraction

Total RNA was extracted from PBMCs using TRIzol reagent (Thermo Fisher Scientific, Waltham, MA, USA) according to the manufacturer’s instructions. RNA concentration and purity were determined using a NanoDrop 2000 spectrophotometer (Thermo Fisher Scientific, Waltham, MA, USA). Samples with an A260/A280 ratio of 1.8–2.0 were considered suitable for further analysis. RNA integrity was assessed by electrophoresis on a 1% agarose gel containing 0.5 μg/mL ethidium bromide by evaluating the 28S and 18S rRNA bands. To eliminate genomic DNA contamination, RNA samples were treated with RNase-free DNase I (Thermo Fisher Scientific, Waltham, MA, USA).

### 2.6. Reverse Transcription and qRT-PCR for miRNA

Reverse transcription was performed using the TaqMan™ MicroRNA Reverse Transcription Kit (Thermo Fisher Scientific, Carlsbad, CA, USA) according to the manufacturer’s protocol. Briefly, 30 ng of total RNA was reverse-transcribed in a final reaction volume of 20 μL using target-specific stem-loop primers for miR-155-5p (Assay ID: 002623), miR-141-3p (Assay ID: 000463), miR-18a-5p (Assay ID: 002422), miR-15b-5p (Assay ID: 000390), and miR-379-5p (Assay ID: 001138) from the TaqMan™ MicroRNA Assay (Thermo Fisher Scientific, Carlsbad, CA, USA). Quantitative real-time PCR (qRT-PCR) was performed in triplicate using qPCRmix-HS Master Mix (Evrogen, Moscow, Russia) on a QuantStudio™ 5 Real-Time PCR System (Applied Biosystems, Thermo Fisher Scientific, Foster City, CA, USA) [12]. The mean Ct value of the three technical replicates was used for quantification. A maximum SD of 0.5 among triplicates was accepted; assays exceeding this threshold were repeated. Relative miRNA expression was normalised to the endogenous control RNU48, whose Ct values were examined across the study groups to assess potential systematic differences in the reference signal. The Ct acceptance range of 20–35 was applied to target miRNAs, whereas values >35 were considered undetectable. RNU48 measurements outside this range were retained for normalisation provided that they satisfied the assay quality-control criteria. Of the 167 participants, valid measurements were available for 160 for miR-155-5p, 159 for miR-141-3p, 159 for miR-15b-5p, 160 for miR-18a-5p and 158 for miR-379-5p. Samples were analysed across multiple plates and runs, with pooled control cDNA included on each plate as an inter-run calibrator. Each run included positive and negative controls, including no-template controls, to verify assay performance and specificity. qRT-PCR procedures and quality control were performed in accordance with the MIQE recommendations [13]. A detailed qRT-PCR protocol has been published previously [12].

### 2.7. RNA Expression Quantification

Ct values were obtained using QuantStudio™ Design & Analysis Software v1.5.2 (Applied Biosystems, Thermo Fisher Scientific, Waltham, MA, USA). Relative miRNA expression was calculated using the comparative ΔΔCt method, where ΔCt = Ct(target) − Ct(reference), and expressed as 2^−ΔΔCt^ [14]. For statistical analysis, relative expression values were log_10_-transformed and presented as median with interquartile range (Me [Q1; Q3]). Fold change (FC) between patients with T2D and controls was calculated as 10^(median T2D−median control)^.

### 2.8. Target Identification and Pathway Enrichment Analysis

In silico target identification and functional enrichment analyses were performed using DIANA-miRPath v4.0 (accessed 22 July 2026) for the four miRNAs that were differentially expressed between participants with T2D and controls. Experimentally supported miRNA–target interactions were obtained from TarBase v8.0, and predicted targets were identified using microT-CDS. KEGG pathway enrichment was assessed separately using TarBase Direct interactions, the broader set of validated TarBase interactions, and microT-CDS predictions at score thresholds of 0.7 and 0.8. Target genes and enriched pathways were analysed using the gene- and pathway-union options implemented in DIANA-miRPath. The number of target genes associated with each enriched pathway was obtained from the DIANA-miRPath output and is reported in Table S7. No custom background gene set was specified for the enrichment analyses. Statistical significance was determined using the enrichment tests implemented by the platform, with Benjamini–Hochberg false discovery rate (FDR) correction for multiple testing, and an FDR-adjusted *p* < 0.05 considered statistically significant.

Pathways significantly enriched in the TarBase Direct analysis and in both microT-CDS analyses were considered consistently enriched across the primary and sensitivity analyses. Of the pathways meeting this criterion, six pathways with relevance to metabolic regulation and T2D (MAPK, Hippo, FoxO, insulin, AMPK and TGF-β signalling) were selected for further examination. Experimentally supported TarBase Direct targets within these pathways were used to assess overlap in target genes across pathways, the contribution of individual miRNAs to miRNA-target interactions, and target genes shared across multiple selected pathways.

### 2.9. Statistical Analysis

Statistical analyses were performed using Microsoft Excel 2021, GraphPad Prism version 9 (GraphPad Software, San Diego, CA, USA), IBM SPSS Statistics version 26.0 (IBM Corp., Armonk, NY, USA), and R version 4.5.2 (R Foundation for Statistical Computing, Vienna, Austria). The distribution of continuous variables was assessed using the Shapiro-Wilk test. Continuous data are presented as median with interquartile range (IQR), whereas categorical variables are expressed as number (%). Between-group comparisons were undertaken using Student’s *t*-test or the Mann–Whitney U test, as appropriate, whilst categorical variables were compared using Pearson’s χ^2^ test or Fisher’s exact test. *p*-values < 0.05 were considered statistically significant. Correction for multiple testing was performed by the Benjamini-Hochberg false discovery rate (FDR) procedure.

Relative miRNA expression values (2^−ΔΔCt^) were log_10_-transformed prior to analysis. Differential expression between patients with T2D and non-diabetic controls was assessed using the Mann–Whitney U test, and associations between miRNA expression and clinical variables were examined using Spearman’s rank correlation coefficient. Fold change was calculated as 10 raised to the difference between the median log10-transformed expression values of the T2D and control groups. Associations between individual miRNAs and T2D were further examined using logistic regression adjusted for age and sex, and additionally for BMI. For these analyses, log10-transformed miRNA expression values were standardised within each corresponding complete-case dataset, and odds ratios were expressed per one-standard-deviation increase in expression.

The ability of individual miRNAs to distinguish between patients with T2D and controls was evaluated by receiver operating characteristic (ROC) curve analysis. Areas under the curve (AUCs) with 95% confidence intervals (CIs), as well as exploratory estimates of sensitivity, specificity, and optimal cut-off values, were calculated for each ROC analysis. Multivariable logistic regression was used to evaluate models containing age, age and sex, and age, sex, and BMI, as well as models containing the four differentially expressed miRNAs (miR-155-5p, miR-15b-5p, miR-18a-5p and miR-379-5p) alone, the four miRNAs with age, and the four miRNAs with age, sex, and BMI. Log10-transformed miRNA expression values were entered into the combined ROC models without standardisation. All models used for direct comparisons were fitted to the same complete-case dataset. Differences between AUCs were assessed using paired DeLong tests. Likelihood-ratio tests were additionally used to assess the contribution of the miRNAs to the corresponding nested logistic regression models. Internal validation was performed using 1000 bootstrap samples generated by random sampling with replacement from the original dataset. The regression model was refitted in each bootstrap sample, and its performance was evaluated both in that sample and in the original dataset. The mean difference between these estimates was used to quantify optimism and was subtracted from the apparent AUC to obtain the optimism-corrected AUC. The four miRNAs were selected based on the differential-expression analysis in the original dataset; miRNA selection was not repeated within the bootstrap samples. For the model containing age, sex, BMI, and the four differentially expressed miRNAs, overfitting of the predicted effects was additionally assessed using the calibration slope. The calibration slope was estimated by logistic regression of T2D status on the model linear predictor; a slope of 1 indicates appropriately scaled predicted effects, whereas a slope below 1 indicates that the predicted effects are too extreme. Optimism in the calibration slope was estimated using the same bootstrap procedure and subtracted from the apparent slope to obtain the optimism-corrected calibration slope.

As a sensitivity analysis to address baseline differences in age, sex, and BMI between participants with T2D and controls, propensity-score overlap weighting was performed. The analysis was restricted to participants with complete data for age, sex, and BMI. Propensity scores for T2D status were estimated using logistic regression including age, sex, and BMI. Overlap weights were assigned as 1 − propensity score for participants with T2D and as the propensity score for controls. Covariate balance before and after weighting was assessed using absolute standardised mean differences (SMDs), with an SMD < 0.10 indicating adequate balance. Effective sample size was calculated separately for each group as the squared sum of the overlap weights divided by the sum of their squares. Associations between T2D status and the four differentially expressed miRNAs were then re-estimated in the overlap-weighted sample using log2-transformed expression values. Heteroskedasticity-consistent (HC3) standard errors were used for statistical inference in the weighted models. *p*-values were adjusted across the four miRNAs using the Benjamini-Hochberg FDR procedure.

## 3. Results

### 3.1. Identification of Candidate miRNAs

We analysed publicly available GEO datasets to assess miRNA expression in PBMCs (GSE75026) [15] and differential miRNA expression in T2D (GSE21321) [8]. We also reviewed the literature to identify miRNAs associated with T2D and its complications (Table S1). Based on these findings, five candidate miRNAs were selected for experimental investigation: miR-155-5p, miR-141-3p, miR-15b-5p, miR-18a-5p, and miR-379-5p.

### 3.2. Clinical Characteristics of the Study Groups

This study included 79 patients with T2D and 88 non-diabetic controls. The groups did not significantly differ regarding sex, but patients with T2D were significantly older than controls (median 56.07 [50.0–66.0] vs. 46.75 [36.0–57.5] years, *p* < 0.001) and had significantly higher BMI, HbA1c, fasting glucose, total cholesterol, LDL cholesterol, and triglyceride concentrations (Table 1).

### 3.3. Differential Expression of Selected miRNAs in Peripheral Blood Mononuclear Cells

**Figure 1:**
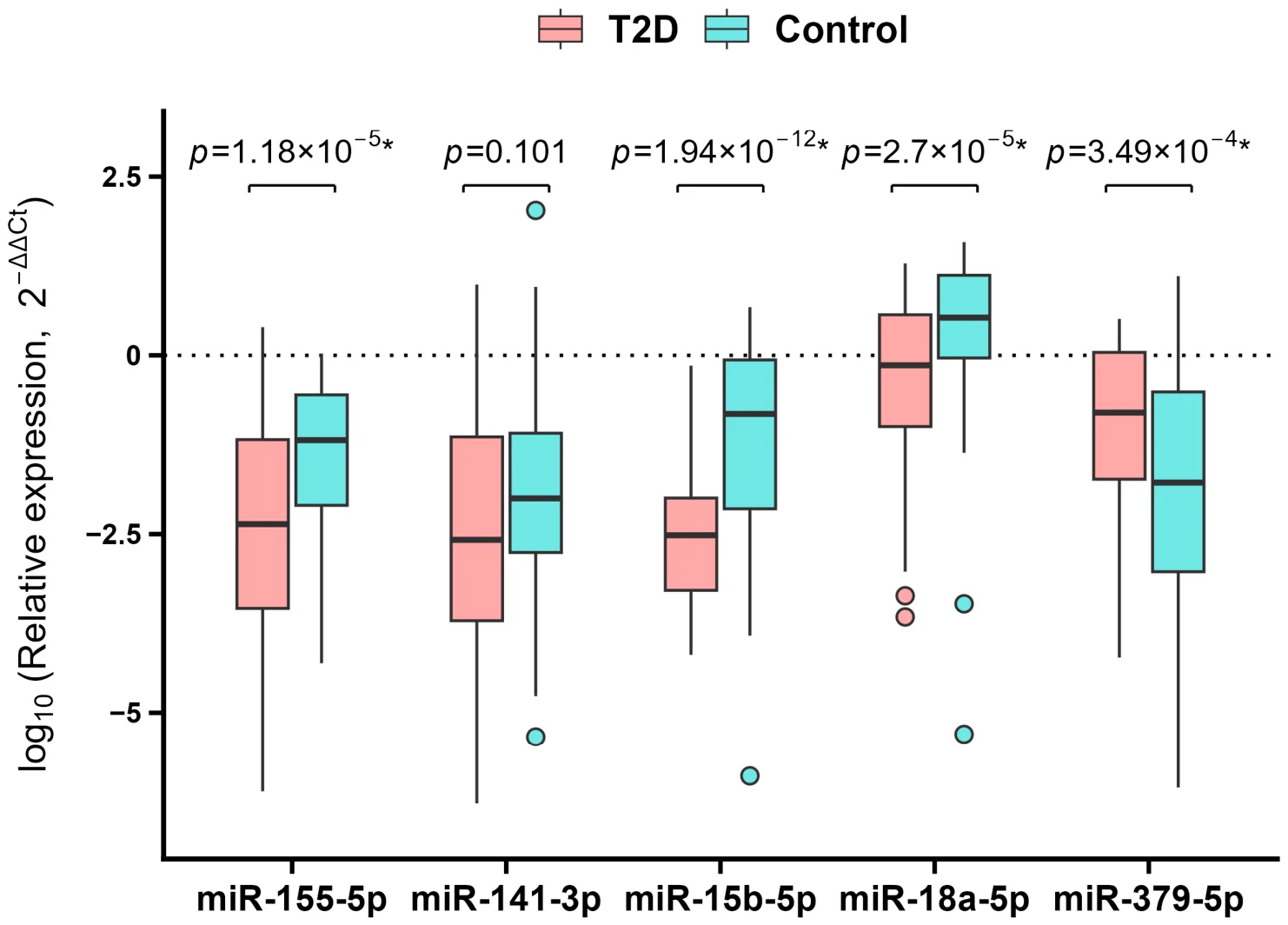
Relative expression of selected miRNAs in peripheral blood mononuclear cells from patients with type 2 diabetes (T2D) and non-diabetic controls. Data are presented as log_10_-transformed relative expression (2^−ΔΔCt^). Boxes represent the interquartile range (IQR), the horizontal line indicates the median, whiskers extend to 1.5 × IQR, and circles represent individual observations. *p*-values were calculated using the Mann–Whitney U test. Statistically significant differences (*p* < 0.05) are indicated by an asterisk (*).

RNU48 Ct values did not differ between the T2D and control groups (*p* = 0.701; Figure S7). Expression analysis demonstrated significant dysregulation of four of the five investigated miRNAs in PBMCs from patients with T2D compared with non-diabetic controls (Table 2, Figure 1). MiR-15b-5p showed the greatest reduction in expression (FC = 0.02, *p* = 1.94 × 10^−12^, *p*_FDR_ = 9.72 × 10^−12^), followed by miR-155-5p (FC = 0.067, *p* = 1.18 × 10^−5^, *p*_FDR_ = 2.94 × 10^−5^) and miR-18a-5p (FC = 0.22, *p* = 2.70 × 10^−5^, *p*_FDR_ = 4.49 × 10^−5^). In contrast, miR-379-5p was significantly upregulated in T2D (FC = 9.53, *p* = 3.49 × 10^−4^, *p*_FDR_ = 4.37 × 10^−4^). No significant difference was observed for miR-141-3p (FC = 0.26, *p* = 0.101).

The associations between individual miRNAs and T2D were further examined after adjustment for age, sex, and BMI (Table 3). Odds ratios were calculated per one-standard-deviation increase in log10-transformed relative miRNA expression. MiR-155-5p, miR-15b-5p and miR-379-5p remained significantly associated with T2D after adjustment, whereas the association with miR-18a-5p was attenuated.

The overlap-weighting sensitivity analysis included 81 controls and 60 participants with T2D who had complete data for age, sex, and BMI. The corresponding effective sample sizes after weighting were 29.23 and 31.34, respectively. Differences in age, sex, and BMI between the groups were reduced to absolute SMDs < 0.01 (Figure S3). The associations with T2D remained significant for miR-155-5p, miR-15b-5p and miR-379-5p after FDR correction, while miR-18a-5p had an FDR-adjusted *p*-value of 0.050 (Table S2).

### 3.4. Associations Between miRNA Expression and Clinical Variables

Correlations between miRNA expression and clinical variables in patients with T2D and controls are summarised in Tables S3 and S4, respectively. Among patients with T2D, the significant clinical correlations involve different aspects of the metabolic phenotype, with distinct patterns among the investigated miRNAs. MiR-141-3p was associated with lipid parameters, correlating negatively with triglycerides and positively with HDL cholesterol (r = −0.373 and 0.464, respectively; *p* ≤ 0.001), whereas miR-15b-5p was positively correlated with fasting glucose and negatively correlated with C-peptide (r = 0.528 and −0.391, respectively; *p* < 0.05). MiR-18a-5p was associated with both HDL cholesterol and C-peptide (r = −0.679 and 0.371, respectively; *p* < 0.05), while miR-155-5p was positively correlated with BMI (r = 0.231, *p* = 0.046). No significant correlations with clinical variables were observed for miR-379-5p. In controls, the only significant association with the examined clinical variables was between miR-15b-5p and fasting glucose (r = 0.279, *p* = 0.039) (Table S4).

Associations between miRNA expression and T2D complications are summarised in Table S5. MiR-15b-5p showed a consistent association with several complications, with higher expression in patients with diabetic nephropathy, diabetic neuropathy, and arterial hypertension (log2FC = 3.04 to 3.33, *p*_FDR_ ≤ 0.002). Association between miR-15b-5p expression and retinopathy was nominally significant but did not survive the FDR correction (*p* = 0.012, *p*_FDR_ = 0.058). No significant associations were observed for the remaining miRNAs after correction for multiple testing.

### 3.5. Discrimination Between T2D and Controls

ROC analysis demonstrated that miR-15b-5p had the highest AUC among the individual miRNAs (AUC = 0.82, 95% CI 0.76–0.89), followed by miR-155-5p (AUC = 0.70, 95% CI 0.62–0.78), miR-18a-5p (AUC = 0.69, 95% CI 0.61–0.77) and miR-379-5p (AUC = 0.66, 95% CI 0.58–0.75) (Figure S1, Table 4). MiR-141-3p did not distinguish between patients with T2D and controls (AUC = 0.58, 95% CI 0.48–0.67) (Figure S1, Table 4).

**Figure 2:**
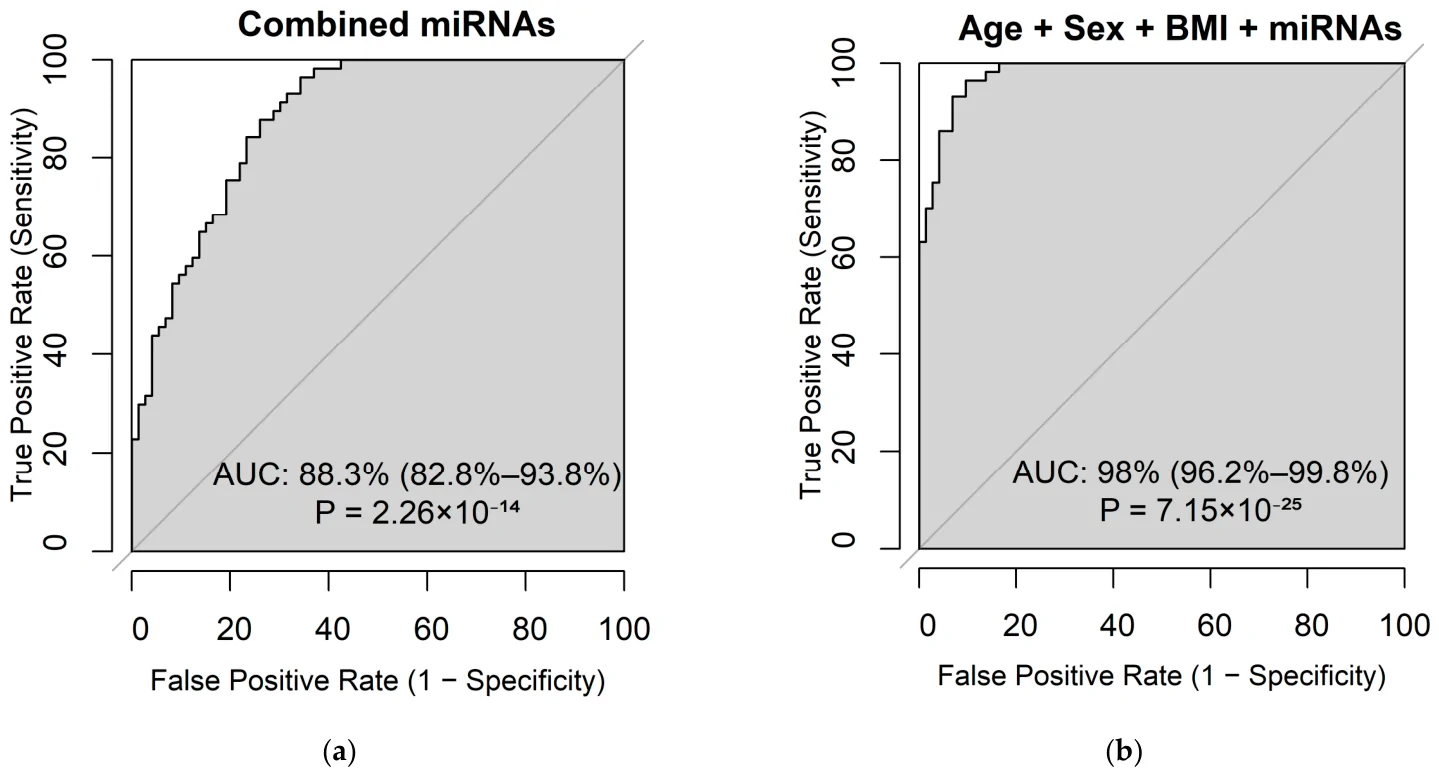
Receiver operating characteristic (ROC) analysis of the combined models. (**a**) Model combining the differentially expressed miRNAs miR-155-5p, miR-15b-5p, miR-18a-5p, and miR-379-5p. (**b**) Model combining the differentially expressed miRNAs with age, sex, and body mass index. AUC, area under the curve.

Combining the four differentially expressed miRNAs increased the AUC to 0.88 (95% CI 0.83–0.94) (Figure 2a). To assess whether the miRNAs improved the ability to distinguish between T2D and controls beyond clinical variables, models incorporating age, sex, and BMI were compared using the same complete-case dataset. The model including age, sex, and BMI had an AUC of 0.93 (Figure S2), which increased to 0.98 following addition of the four differentially expressed miRNAs (*p* = 0.004, paired DeLong test) (Figure 2b). The addition of the four miRNAs also significantly improved model fit (likelihood-ratio test: χ^2^(4) = 37.34, *p* = 1.53 × 10^−7^). Bootstrap internal validation yielded an optimism-corrected AUC of 0.97 and an optimism-corrected calibration slope of 0.72 for the model containing age, sex, BMI, and the four miRNAs. However, miRNA selection was not repeated within the bootstrap samples; therefore, the optimism-corrected estimates do not account for optimism arising from feature selection. Regression coefficients, 95% CIs, and *p*-values for this model are presented in Table S6.

### 3.6. Functional Enrichment Analysis of miRNA Targets

**Figure 3:**
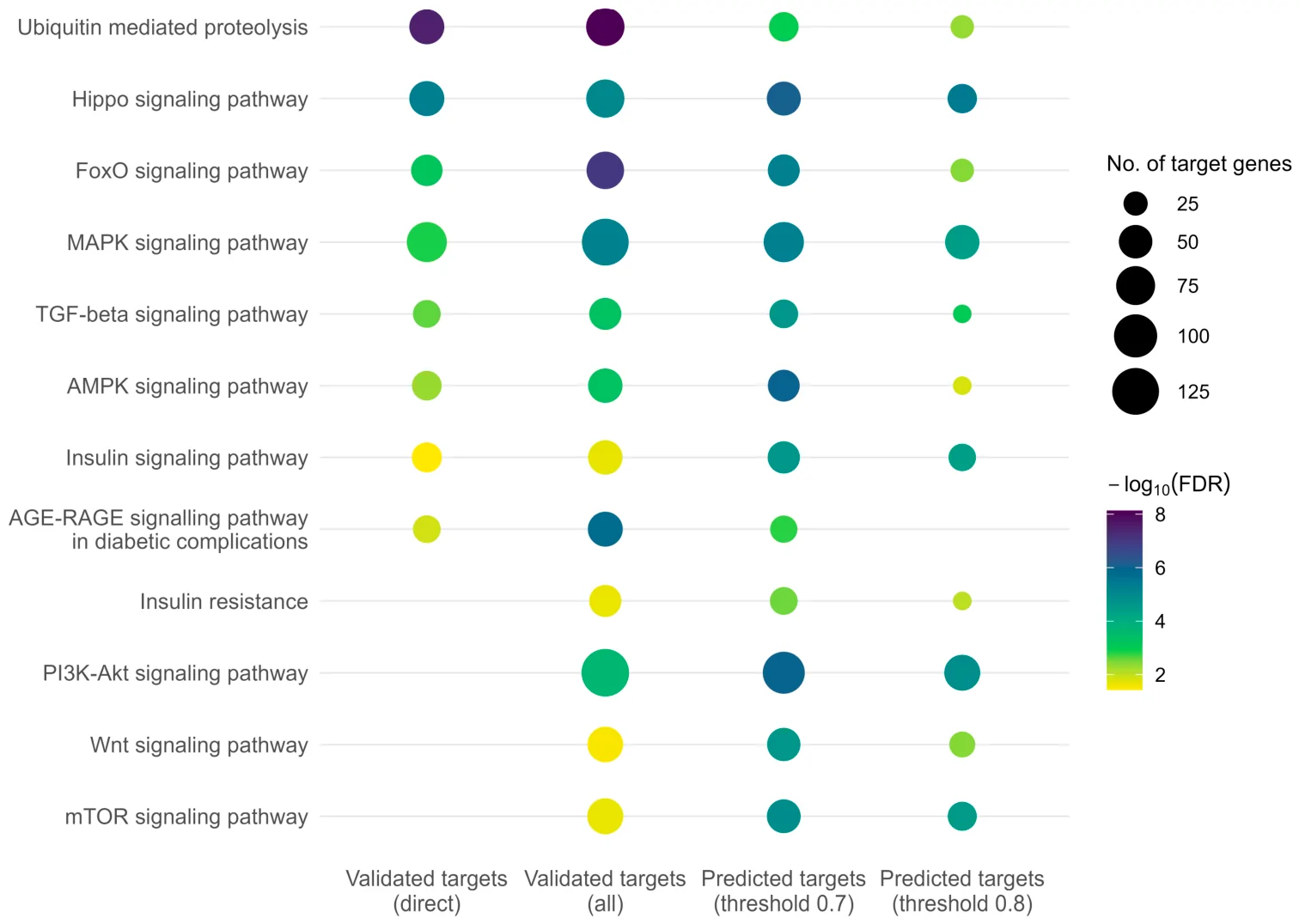
KEGG pathway enrichment across miRNA target-analysis approaches. Bubble size represents the number of target genes assigned to each pathway, and colour represents enrichment significance as −log10(FDR). Columns show analyses based on TarBase Direct experimentally supported targets, the full TarBase validated-target set, and microT-CDS predicted targets at thresholds of 0.7 and 0.8. Blank cells indicate that the pathway did not meet the significance criterion in the corresponding analysis.

Functional enrichment analysis of the four differentially expressed miRNAs identified multiple significantly enriched KEGG pathways, with several pathways recurring across analyses based on experimentally supported and predicted mRNA targets (Table S7; Figure 3). MAPK, Hippo, FoxO, insulin, AMPK, and TGF-β signalling were consistently enriched in the TarBase Direct analysis and both microT-CDS analyses and were selected for further examination.

Considerable overlap was observed among the TarBase Direct target genes assigned to these six pathways (Figure S4). Analysis of the contributions of individual miRNAs showed that miR-15b-5p accounted for the largest number of experimentally supported miRNA–target interactions across the selected pathways, with additional contributions from miR-155-5p and miR-18a-5p and comparatively few interactions involving miR-379-5p (Figure S5). Several target genes were shared across three or more of the selected pathways, including genes involved in MAPK, FoxO, insulin and AMPK signalling (Table S8, Figure S6).

## 4. Discussion

We evaluated the expression of five miRNAs in PBMCs of patients with T2D and non-diabetic controls, and found downregulation of miR-155-5p, miR-15b-5p, and miR-18a-5p, and upregulation of miR-379-5p. The associations of miR-155-5p, miR-15b-5p, and miR-379-5p with T2D remained significant after adjustment for age, sex, and BMI. MiR-15b-5p showed the largest difference in expression between patients and controls and the highest AUC among the individual miRNAs (0.82). The four differentially expressed miRNAs had an AUC of 0.88 when analysed together. Adding these miRNAs to age, sex, and BMI increased the AUC from 0.93 to 0.98, with improvement also observed in the likelihood-ratio comparison of the nested models. After bootstrap correction for optimism, the combined model had an AUC of 0.97 and a calibration slope of 0.72. Functional enrichment analysis identified MAPK, Hippo, FoxO, insulin, AMPK, and TGF-β signalling as consistently enriched across analyses based on TarBase Direct targets and microT-CDS predicted targets.

MiR-15b-5p has previously been found to be expressed differently in various tissues and biological samples in T2D. Increased miR-15b-5p in urinary extracellular vesicles has been associated with albuminuria, HbA1c, blood pressure, creatinine concentration, and accelerated decline in estimated glomerular filtration rate (eGFR) [16,17,18,19,20,21,22,23,24]. In contrast, reduced expression has been reported in mesangial cells exposed to high glucose and in diabetic nephropathy, where restoration of miR-15b-5p reduced extracellular matrix accumulation, inflammation, and WNT2B signalling [20]. Altered miR-15b-5p expression has also been described in diabetic wounds, retinal endothelial cells, and skeletal muscle [18,19,20,21,22,23,24].

MiR-15b-5p has been implicated in apoptosis, proliferation, insulin signalling, and inflammatory responses [23,24], as well as obesity, T2D, and diabetic microvascular complications [23,24,25]. In our study, MAPK, Hippo, FoxO, insulin, AMPK, and TGF-β signalling were consistently enriched across analyses based on experimentally supported and predicted targets of the differentially expressed miRNAs. Among the experimentally supported interactions within these pathways, miR-15b-5p accounted for the largest number of miRNA–target interactions. FoxO, insulin, and AMPK signalling were also identified among predicted miR-15b-5p targets in a previous study of childhood obesity [26]. These enrichment and target-overlap analyses provide biological context for the observed miRNA expression differences but do not establish altered pathway activity in PBMCs.

Previously, inconsistent changes in miR-155-5p expression in T2D have been reported, which may partly reflect differences in the biological material analysed and the characteristics of the study populations [27,28,29,30,31,32,33,34,35,36,37,38]. We detected that miR-155-5p was significantly downregulated in PBMCs, consistent with previous reports in PBMCs and platelets [27,28,29], as well as studies describing reduced expression in gestational diabetes and insulin resistance [33,38]. Given the central role of PBMCs in chronic low-grade inflammation, decreased miR-155-5p expression may reflect altered immune-cell activation associated with T2D.

MiR-155-5p has also been implicated in the regulation of metabolic and inflammatory processes. Experimental studies have shown that miR-155 deficiency impairs β-cell expansion during metabolic stress, reduces insulin secretion, increases glucagon production, and alters islet cell composition, while having limited effects on islet morphology under basal conditions [35,39]. MiR-155-5p expression is regulated by LDL through MafB and is involved in immune and inflammatory signalling [39]. These findings suggest that reduced PBMC miR-155-5p expression may be linked to dysregulation of metabolic and inflammatory pathways in T2D, although the underlying mechanisms remain incompletely understood [40,41,42,43,44,45].

MiR-18a-5p was also significantly downregulated in PBMCs from patients with T2D. Previous studies have associated miR-18a-5p with T2D, diabetic nephropathy, and insulin sensitivity, although reported expression patterns have varied across biological samples and populations [46,47,48,49]. In addition to diabetes, miR-18a-5p has been linked to cardiovascular disease through the regulation of cardiomyocyte autophagy and apoptosis mediated by oxidative stress [50,51,52].

MiR-379-5p was significantly upregulated in our study group. Previously, it has been reported as both upregulated and downregulated in T2D and its complications [53,54], potentially reflecting differences in sample type, disease stage, clinical characteristics, and analytical methods. MiR-379-5p regulates coding and non-coding RNAs involved in metabolic homeostasis and diabetic complications [53,55], suggesting that its functional effects may also be context-dependent [55,56,57,58].

The differentially expressed miRNAs showed greater discrimination when analysed together than individually and provided additional discriminatory information when added to age, sex and BMI. The increase in AUC from 0.93 to 0.98 was supported by the paired DeLong test and likelihood-ratio comparison, although the performance of the combined model requires confirmation in an independent cohort.

### Study Limitations and Future Directions

This study has several limitations. The findings are based on a single-centre cohort of modest size, which may limit their generalisability. In addition, the cross-sectional study design does not allow assessment of how miRNA expression changes over the course of disease or whether the observed associations reflect causal mechanisms. Although the enrichment analyses identified pathways consistently across experimentally supported and predicted miRNA targets, these findings do not demonstrate altered pathway activity and require functional validation. An important limitation is the substantial baseline difference in age and BMI between the T2D and control groups, which introduces potential confounding in the primary case-control comparisons. We therefore performed an overlap-weighted sensitivity analysis in participants with complete age, sex, and BMI data. Weighting achieved balance in these measured covariates, and the direction of association for all four differentially expressed miRNAs was preserved. However, the limited covariate overlap and reduced effective sample sizes restrict the generalisability of the weighted estimates and do not exclude residual confounding by other factors. Information on medication use, smoking status, and inflammatory comorbidities was incomplete or unavailable and could not be accounted for in the analyses; these factors may have influenced PBMC miRNA expression. PBMC subpopulations were not assessed, and differences in cellular composition may also have contributed to the observed miRNA expression differences. The discriminatory analyses were conducted in the same case-control cohort in which the differentially expressed miRNAs were identified; although bootstrap correction was applied, miRNA selection was not repeated within the bootstrap procedure. Confirmation in independent cohorts with more comparable baseline characteristics is therefore necessary. Finally, because miRNA expression is tissue-specific, the PBMC expression changes described here may not reflect molecular changes occurring in other tissues involved in T2D. Future studies incorporating independent validation, longitudinal follow-up, and functional analyses will be important to confirm these findings and further define the role of these miRNAs in T2D.

## 5. Conclusions

In our study, we identified differential PBMC expression of four selected miRNAs in T2D, with downregulated miR-15b-5p, miR-155-5p, and miR-18a-5p, and upregulated miR-379-5p. Functional enrichment analysis identified MAPK, Hippo, FoxO, insulin, AMPK, and TGF-β signalling as consistently enriched across analyses based on experimentally supported and predicted miRNA targets. The differentially expressed miRNAs also improved discrimination between patients with T2D and non-diabetic controls when added to age, sex, and BMI, with the combined model achieving an AUC of 0.98. These findings support further investigation of PBMC miRNAs as potential molecular markers of T2D and of the functional relationships suggested by their target and pathway enrichment analyses. Validation in independent cohorts and functional studies will be required to confirm their discriminatory performance and determine the biological significance of the identified miRNA-target relationships.

## Figures and Tables

**Table 1:** Clinical characteristics of the study participants.

Parameter	Control (*n* = 88) *	T2D (*n* = 79) *	*p*	*p*_FDR_
Age, years	46.75 (36–57.5)	56.07 (50–66)	<0.001	<0.001
Sex, *n* (%)				
Male	27 (30.68)	29 (36.71)	0.380	0.380
Female	61 (69.32)	50 (63.29)		
BMI, kg/m^2^	22.93 (19.91–26.01)	32.30 (29.43–34.61)	<0.001	3.00 × 10^−4^
Obesity, *n* (%)	–	57 (72.15)	–	
Duration of T2D, years	–	10.00 (3–15)	–	
Arterial hypertension, *n* (%)	–	50 (63.29)	–	
Diabetic nephropathy, *n* (%)	–	58 (73.41)	–	
Diabetic neuropathy, *n* (%)	–	52 (65.82)	–	
Diabetic retinopathy, *n* (%)	–	25 (31.64)	–	
HbA1c (%),	4.90 (3.8–5.60)	9.30 (7.30–14.10)	<0.001	3.00 × 10^−4^
Fasting glucose, mmol/L	4.80 (3.20–5.90)	9.60 (6.95–12.55)	<0.001	3.00 × 10^−4^
Total cholesterol, mmol/L	4.50 (3.30–6.12)	4.94 (4.34–5.57)	0.001	0.002
LDL cholesterol, mmol/L	2.70 (0.79–3.99)	2.98 (1.81–3.85)	<0.005	0.009
HDL cholesterol, mmol/L	1.10 (0.87–1.40)	1.13 (0.71–1.17)	0.080	0.100
Triglycerides, mmol/L	1.32 (1.10–2.07)	2.34 (1.75–3.49)	0.029	0.040

* Continuous variables are presented as median (interquartile range, IQR) and categorical variables as *n* (%). T2D—type 2 diabetes; *p*—*p* value; *p*_FDR_—false discovery rate (FDR)-adjusted *p* value; BMI—body mass index; HbA1c—glycated haemoglobin; LDL–low-density lipoprotein cholesterol; HDL—high-density lipoprotein cholesterol.

**Table 2:** Relative expression of selected miRNAs in peripheral blood mononuclear cells from patients with type 2 diabetes and non-diabetic controls.

miRNA	T2D *	Control *	FC	Regulation	*p*	*p*_FDR_
miR-155-5p	−2.36 [−3.53; −1.17]	−1.18 [−2.09; −0.55]	0.07	↓	1.18 × 10^−5^	2.94 × 10^−5^
miR-141-3p	−2.58 [−3.71; −1.14]	−2.00 [−2.75; −1.08]	0.26	↓	0.101	0.101
miR-15b-5p	−2.51 [−3.28; −1.99]	−0.81 [−2.14; −0.06]	0.02	↓	1.94 × 10^−12^	9.72 × 10^−12^
miR-18a-5p	−0.13 [−0.99; 0.56]	0.52 [−0.03; 1.12]	0.22	↓	2.70 × 10^−5^	4.49 × 10^−5^
miR-379-5p	−0.80 [−1.73; 0.04]	−1.77 [−3.02; −0.51]	9.53	↑	3.49 × 10^−4^	4.37 × 10^−4^

* T2D—type 2 diabetes; FC—fold change; *p*—*p* value; *p*_FDR_—FDR-adjusted *p* value. Data are presented as median (interquartile range, IQR) of log_10_-transformed relative expression. Group comparisons were performed using the Mann–Whitney U test. *p*-values were adjusted for multiple comparisons using the Benjamini-Hochberg false discovery rate (FDR) procedure. Fold change (FC) was calculated as 10^(median T2D−median control)^. Arrows indicate the direction of differential expression (↑, upregulated; ↓, downregulated).

**Table 3:** Associations between individual miRNAs and T2D with adjustment for clinical parameters.

miRNA	Model	N	Adjusted OR (95% CI)	*p*	*p*_FDR_
miR-155-5p	Age + Sex	134	0.48 (0.28–0.80)	0.006	0.01
	Age + Sex + BMI	134	0.49 (0.26–0.84)	0.014	0.024
miR-141-3p	Age + Sex	133	0.87 (0.54–1.37)	0.541	0.541
	Age + Sex + BMI	133	0.79 (0.46–1.31)	0.367	0.367
miR-15b-5p	Age + Sex	133	0.33 (0.18–0.55)	1.04 × 10^−4^	5.20 × 10^−4^
	Age + Sex + BMI	133	0.36 (0.19–0.65)	0.001	0.006
miR-18a-5p	Age + Sex	134	0.61 (0.37–0.95)	0.034	0.042
	Age + Sex + BMI	134	0.64 (0.38–1.02)	0.065	0.081
miR-379-5p	Age + Sex	132	2.36 (1.42–4.24)	0.002	0.005
	Age + Sex + BMI	132	2.69 (1.49–5.41)	0.002	0.006

*p*-values were adjusted for multiple comparisons using the Benjamini-Hochberg false discovery rate (FDR) procedure. OR—odds ratio; 95%CI—95% confidence interval; *p*—*p* value; *p*_FDR_—FDR-adjusted *p* value. ORs are expressed per one-standard-deviation increase in log10-transformed relative miRNA expression (2^−ΔΔCt^). Each miRNA was standardised within its corresponding complete-case analysis dataset.

**Table 4:** Discriminatory performance of selected miRNAs, clinical parameters, and combined models for distinguishing patients with type 2 diabetes from non-diabetic controls.

Biomarker	AUC (95% CI)	Sensitivity (%)	Specificity (%)	Optimal Cut-Off
miR-155-5p	0.70 (0.62–0.78)	51.32	82.14	<−2.34
miR-141-3p	0.58 (0.48–0.67)	32.89	89.16	<−3.45
miR-15b-5p	0.82 (0.76–0.89)	84.21	71.08	<−1.51
miR-18a-5p	0.69 (0.61–0.77)	86.84	42.86	<0.79
miR-379-5p	0.66 (0.58–0.75)	61.84	69.51	>−1.13
Combined miRNAs	0.88 (0.83–0.94)	95.00	68.92	>0.27
Age	0.89 (0.83–0.95)	81.67	82.43	>0.47
Age + Sex	0.89 (0.83–0.95)	81.67	82.43	>0.47
Age + Sex + BMI	0.93 (0.89–0.97)	90.00	85.14	>0.42
miRNAs + Age	0.97 (0.95–0.99)	90.00	94.59	>0.52
miRNAs + Age + Sex + BMI	0.98 (0.96–1.00)	96.67	90.54	>0.26

AUC—area under the receiver operating characteristic curve; CI—confidence interval. For individual miRNAs, cut-offs refer to log10-transformed relative expression values; for combined and clinical models, cut-offs refer to predicted probabilities. Sensitivity, specificity, and cut-off values are exploratory.

## Data Availability

The datasets generated and/or analysed during the current study are available from the corresponding author upon reasonable request.

## Supplementary Materials

The following supporting information can be downloaded at: https://www.mdpi.com/article/10.3390/biom16101503/s1, Figure S1: ROC curves for the studied microRNAs: (a) miR-155-5p, (b) miR-141-3p, (c) miR-15b-5p, (d) miR-18a-5p, and (e) miR-379-5p; Figure S2: ROC curves for models based on clinical characteristics: (a) age, (b) age and sex, and (c) age, sex and body mass index; Figure S3: Covariate balance before and after overlap weighting; Figure S4: Overlap of TarBase Direct target genes across the six selected KEGG pathways; Figure S5: Contributions of individual miRNAs to experimentally supported miRNA–target interactions across the six selected KEGG pathways; Figure S6: Experimentally supported target genes shared across the selected KEGG pathways; Figure S7: RNU48 expression in patients with T2D and non-diabetic controls; Table S1: Dysregulation of selected miRNAs in type 2 diabetes and associated complications reported in previous studies [16,17,18,19,20,22,23,24,25,26,28,29,30,31,32,33,36,39,40,41,46,47,48,49,51,52,53,55,57,58,59,60,61,62,63,64,65,66,67]; Table S2: Overlap-weighted associations between miRNA expression and T2D; Table S3: Correlations between miRNA expression and clinical and biochemical parameters in patients with T2D; Table S4: Correlations between miRNA expression and clinical and biochemical parameters in non-diabetic controls; Table S5: Associations between miRNA expression and clinical complications in patients with T2D; Table S6: Logistic regression coefficients for the combined model including the differentially expressed miRNAs, age, sex and body mass index; Table S7: Complete KEGG pathway enrichment results; Table S8: TarBase Direct target genes in the six selected KEGG pathways.

## Abbreviations

The following abbreviations are used in this manuscript:

AUC	Area under the curve
BMI	Body mass index
CI	Confidence interval
FC	Fold change
FDR	False discovery rate
GEO	Gene Expression Omnibus
HbA1c	Glycated haemoglobin
HDL	High-density lipoprotein
KEGG	Kyoto Encyclopedia of Genes and Genomes
LDL	Low-density lipoprotein
MAPK	Mitogen-activated protein kinase
miRNA	MicroRNA
PBMCs	Peripheral blood mononuclear cells
PI3K	Phosphoinositide 3-kinase
ROC	Receiver operating characteristic
STRING	Search Tool for the Retrieval of Interacting Genes/Proteins
T2D	Type 2 diabetes
