# Supplementary material for: Expression Analysis of microRNAs in Peripheral Blood Mononuclear Cells in Type 2 Diabetes
Source: Biomolecules. 2026 Oct 15;16(10):1503. doi: 10.3390/biom16101503 (PMC13702599; doi:10.3390/biom16101503)
Supplement: Supplementary file 1 [file biomolecules-16-01503-s001.zip › Supplementary_Figures_revised.pdf]

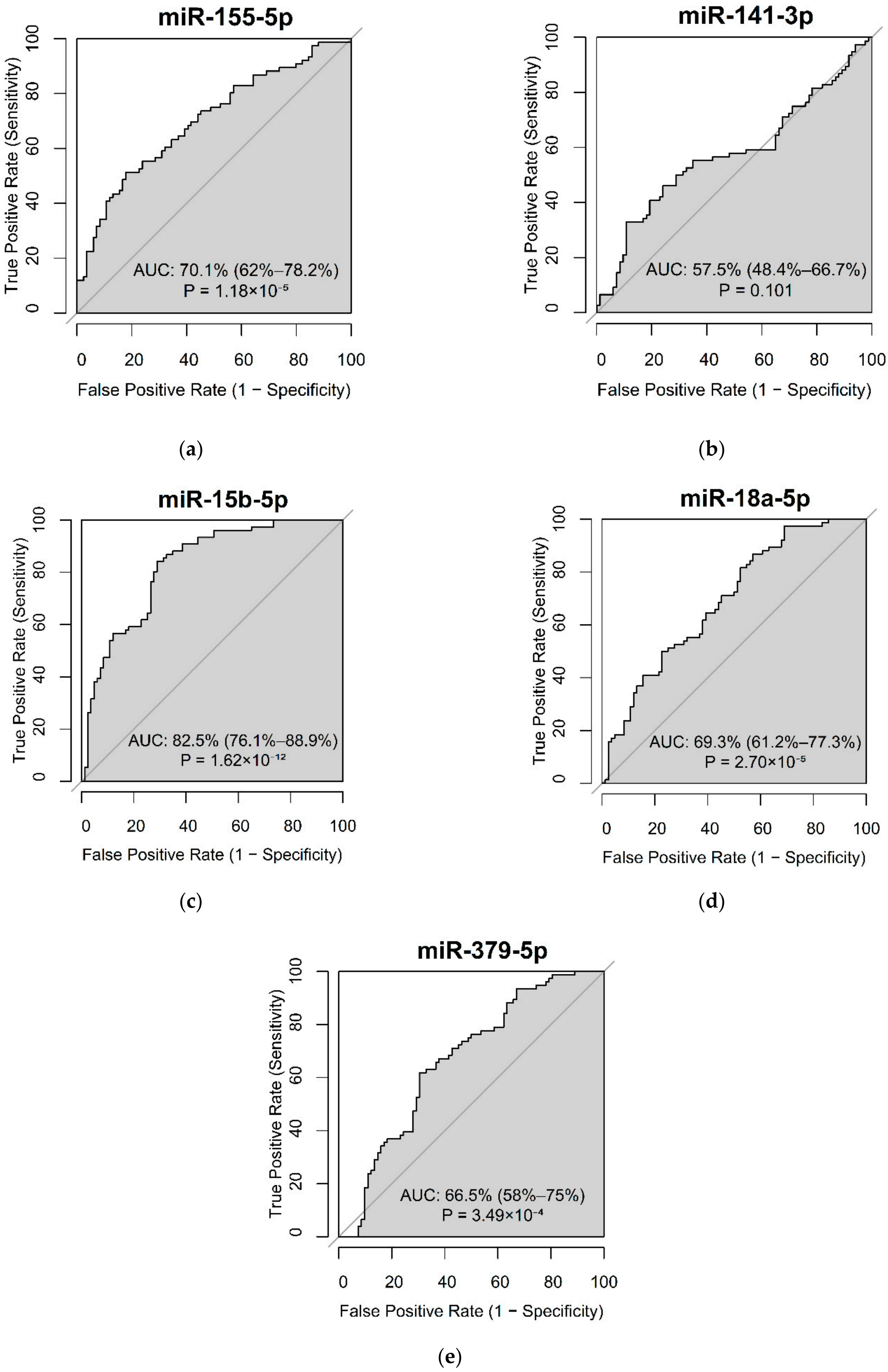

Figure S1. ROC curves for the studied microRNAs: (**a**) miR-155-5p (**b**) miR-141-3p (**c**) miR-15b-5p (**d**) miR-18a-5p (**e**) miR-379-5p.

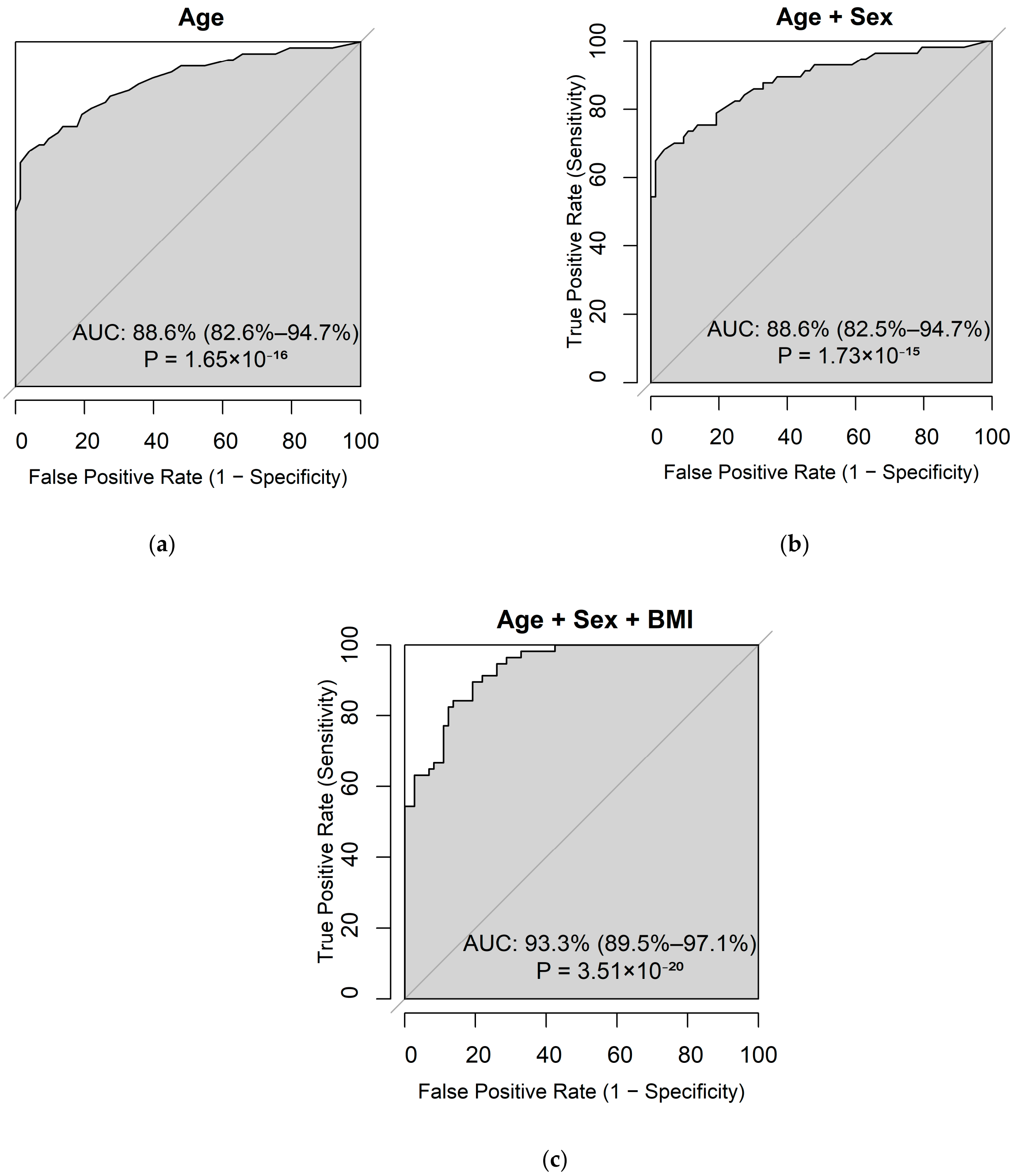

Figure S2. ROC curves for clinical parameters: (**a**) age, (**b**) age + sex, (**c**) age + sex + body mass index.

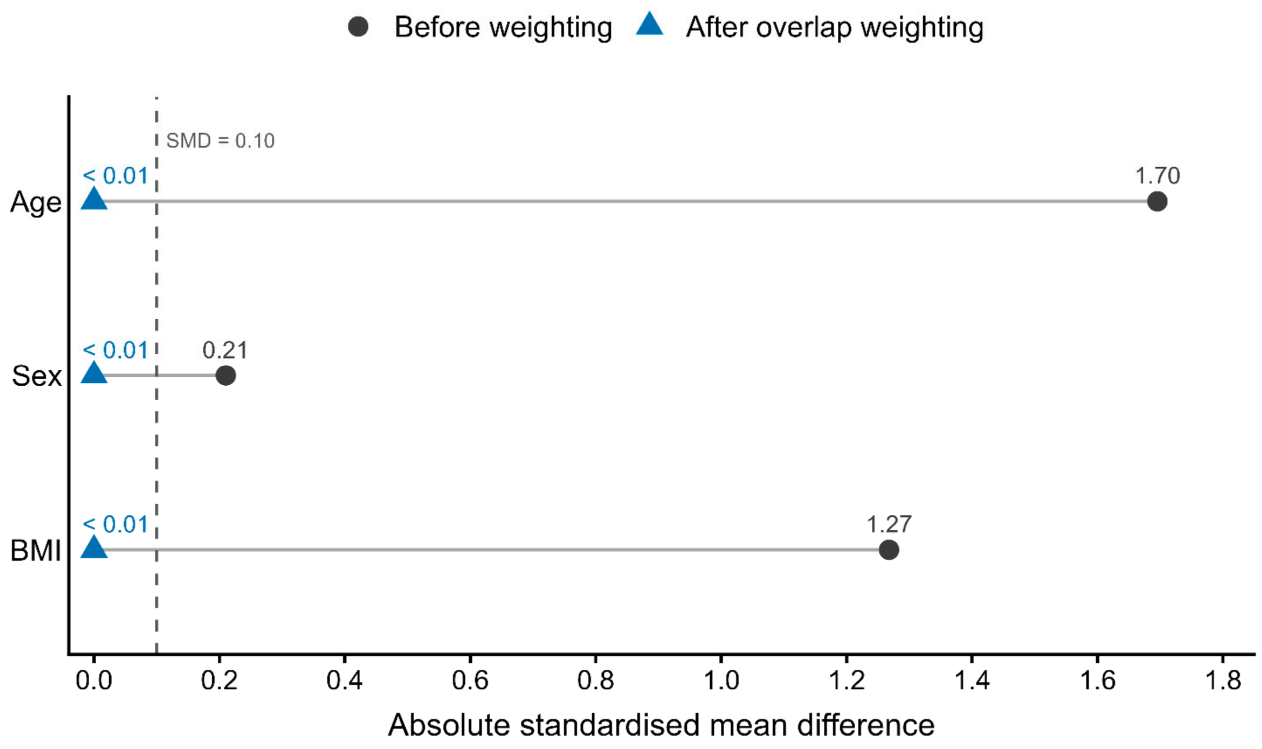

Figure S3. Covariate balance before and after propensity-score overlap weighting.

Absolute standardised mean differences (SMDs) are shown for age, sex, and BMI before weighting and after overlap weighting based on propensity scores estimated from these covariates. The dashed vertical line indicates an absolute SMD of 0.10. Overlap weighting substantially reduced the measured baseline imbalance between participants with T2D and controls.

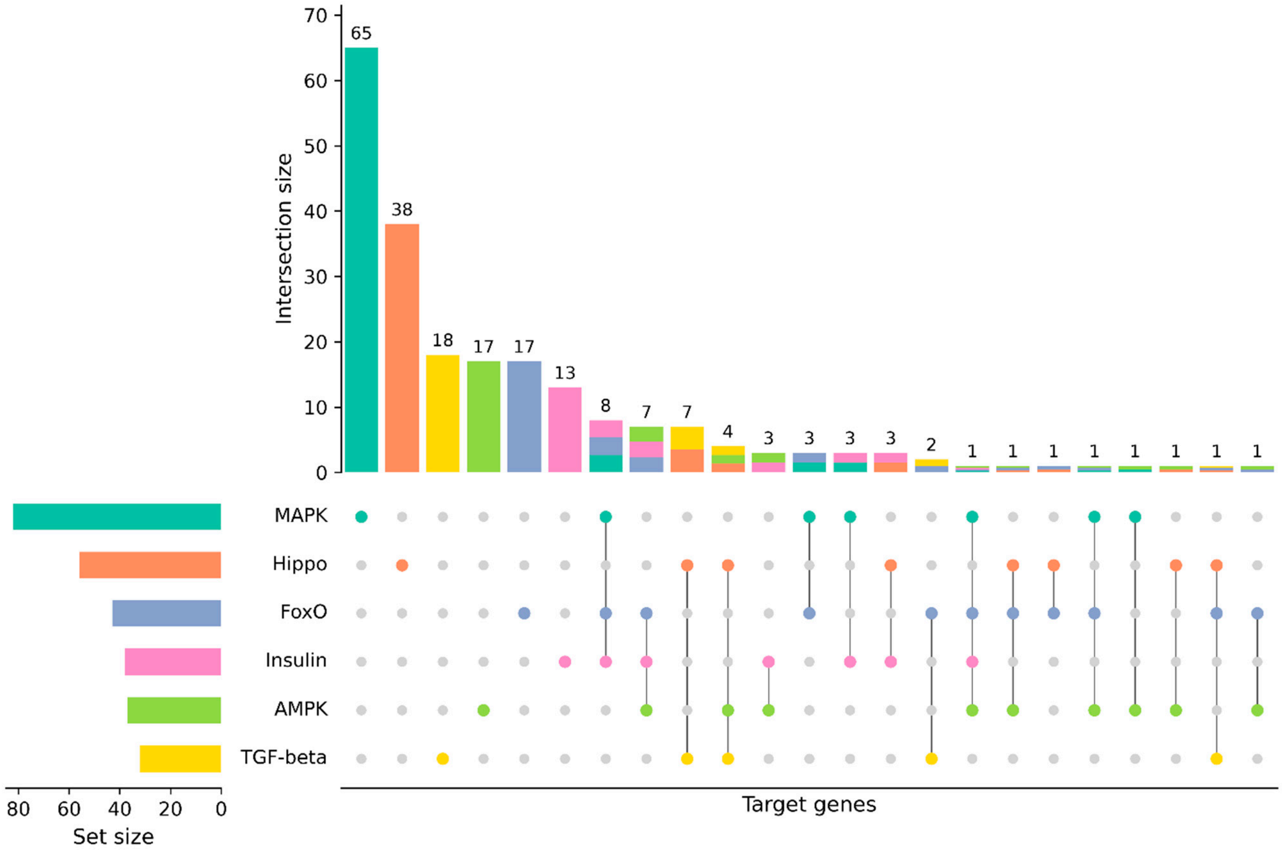

Figure S4. Overlap of TarBase v8 Direct target genes across six selected KEGG pathways.

The horizontal bars show the total number of unique target genes assigned to each pathway, while vertical bars show the sizes of unique and shared gene intersections across pathways.

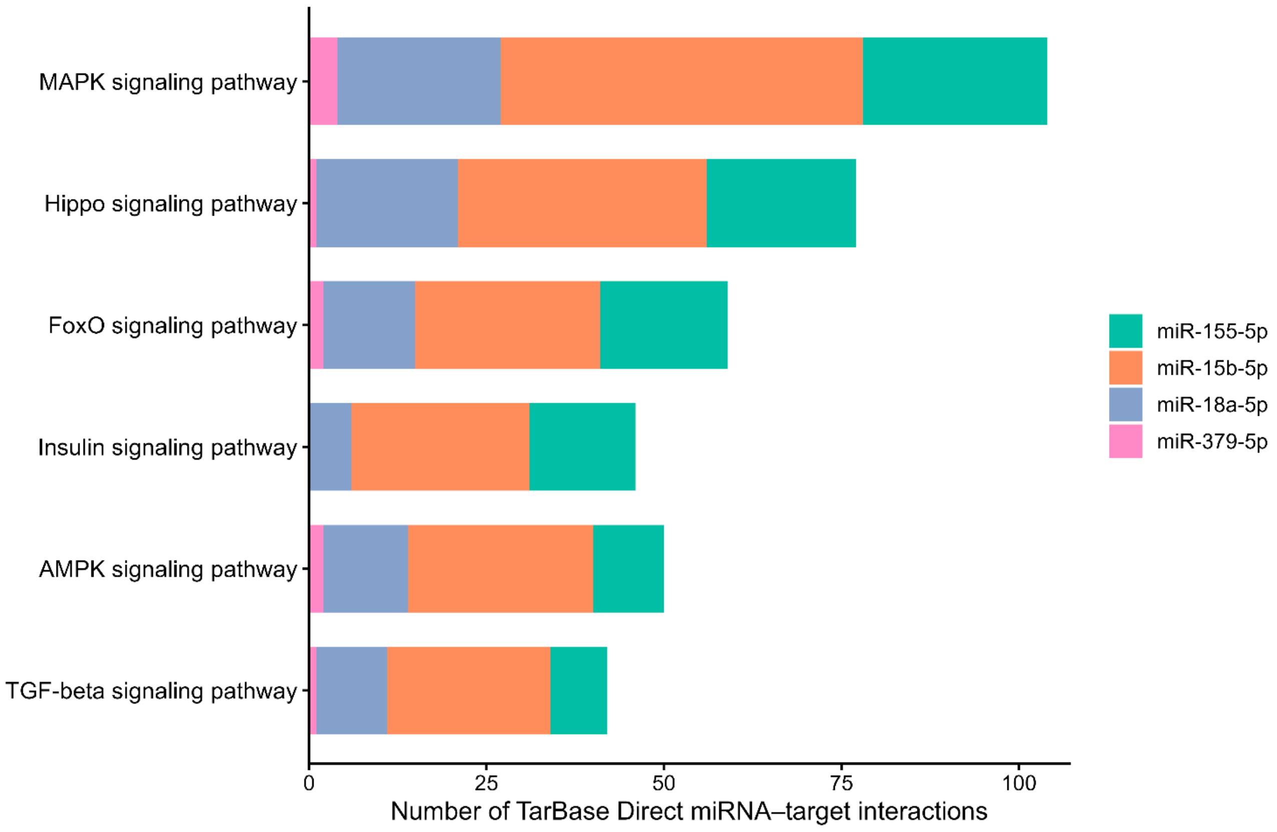

Figure S5. Contribution of individual miRNAs to TarBase Direct interactions within six selected KEGG pathways.

Stacked bars show the number of TarBase Direct miRNA-target interactions involving each of the four differentially expressed miRNAs within six selected pathways that were consistently enriched across the primary and sensitivity analyses. Interaction counts are not equivalent to numbers of unique target genes because the same gene may be targeted by more than one miRNA.

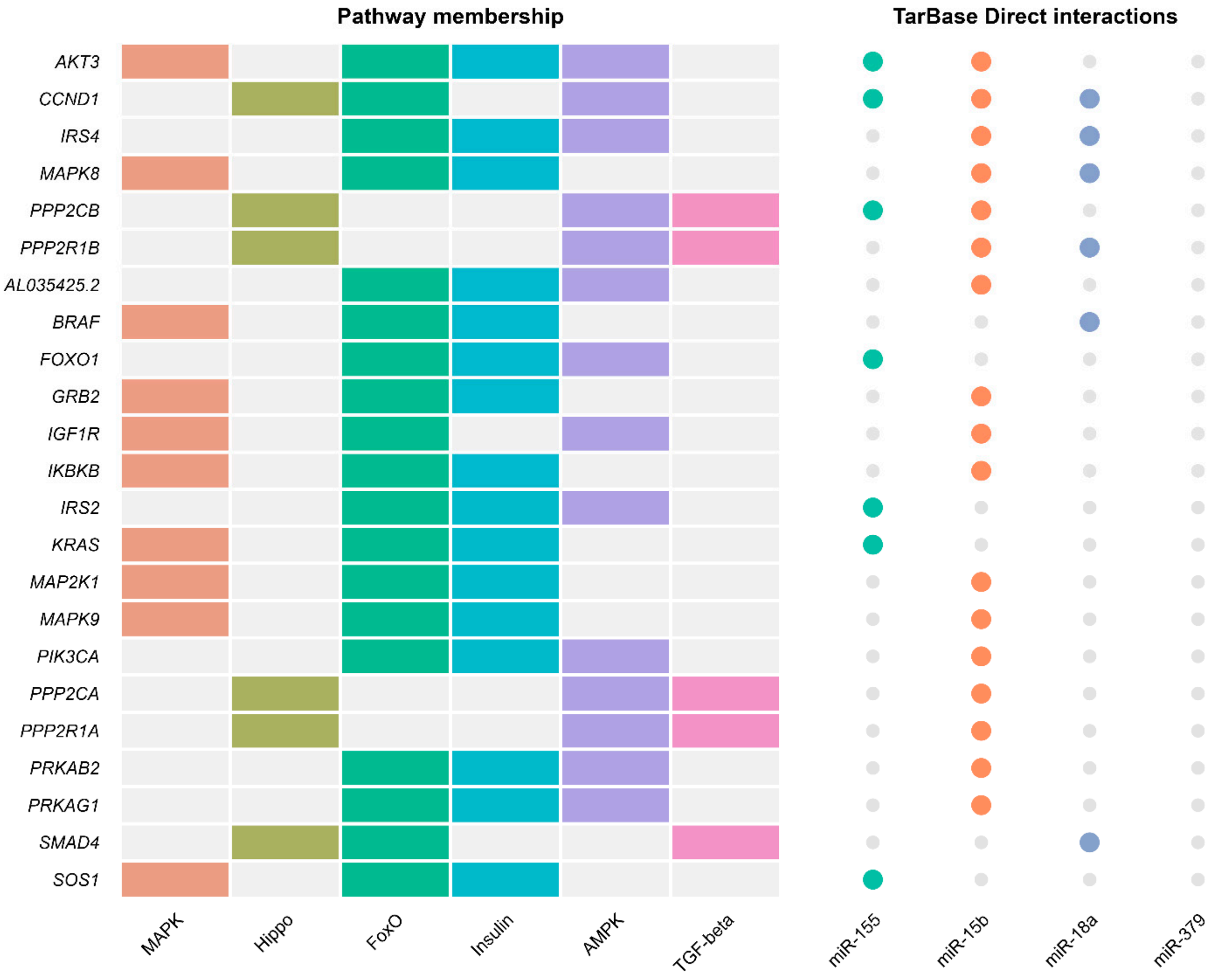

Figure S6. Shared experimentally supported miRNA targets across six selected KEGG pathways.

Genes occurring in at least three of six selected pathways consistently enriched across the primary and sensitivity analyses are shown. The left matrix indicates pathway membership for TarBase v8 Direct target genes, while the right matrix indicates which of the four differentially expressed miRNAs have experimentally supported TarBase Direct interactions with each gene. Filled squares indicate pathway membership; coloured circles indicate miRNA–target interactions. The figure represents database-supported target overlap and does not demonstrate altered expression or activity of these genes or pathways in the study participants

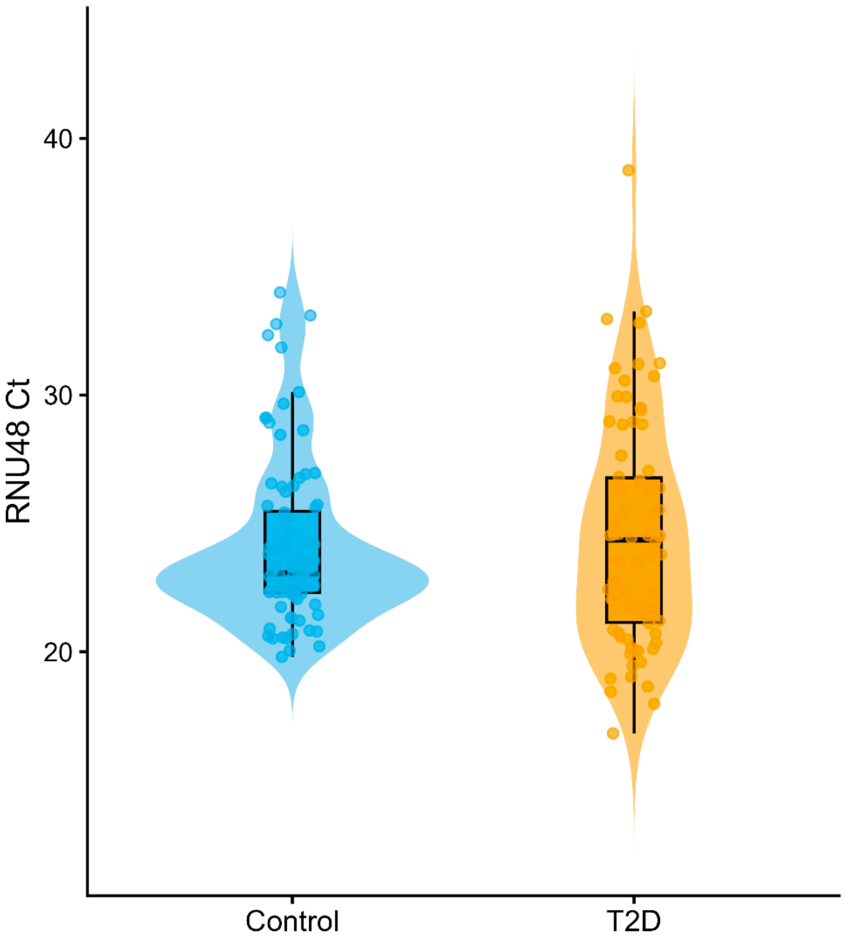

Figure S7. Distribution of RNU48 Ct values in peripheral blood mononuclear cells of controls and participants with type 2 diabetes.

Violin plots show the distribution of raw RNU48 Ct values, with individual observations overlaid. Boxplots indicate the median and interquartile range. RNU48 Ct values were available for 88 controls and 78 participants with T2D. All available RNU48 measurements, including those outside the Ct acceptance range of 20–35 applied to the target miRNAs, were retained for normalisation and included in the figure. No statistically significant between-group difference was observed (Welch's t-test, P = 0.437; Mann–Whitney test, P = 0.701)
